# Potentiation of Schaffer-Collateral CA1 Synaptic Transmission by eEF2K and p38 MAPK Mediated Mechanisms

**DOI:** 10.3389/fncel.2016.00247

**Published:** 2016-10-25

**Authors:** Weiguang Weng, Ying Chen, Man Wang, Yinghan Zhuang, Thomas Behnisch

**Affiliations:** The Institutes of Brain Science, The State Key Laboratory of Medical Neurobiology, and the Collaborative Innovation Center for Brain Science, Fudan UniversityShanghai, China

**Keywords:** hippocampus, protein synthesis, memory, synaptic plasticity, eEF2, MAPK, oscillation

## Abstract

The elongation factor 2 kinase (eEF2K), likewise known as CaMKIII, has been demonstrated to be involved in antidepressant responses of NMDA receptor antagonists. Even so, it remains open whether direct inhibition of eEF2K without altering up-stream or other signaling pathways affects hippocampal synaptic transmission and neuronal network synchrony. Inhibition of eEF2K by the selective and potent eEF2K inhibitor A-484954 induced a fast pre-synaptically mediated enhancement of synaptic transmission and synchronization of neural network activity. The eEF2K-inhibition mediated potentiation of synaptic transmission of hippocampal CA1 neurons is most notably independent of protein synthesis and does not rely on protein kinase C, protein kinase A or mitogen-activated protein kinase (MAPK)/extracellular signal-regulated protein kinase 1/2. Moreover, the strengthening of synaptic transmission in the response to the inhibition of eEF2K was strongly attenuated by the inhibition of p38 MAPK. In addition, we show the involvement of barium-sensitive and more specific the TWIK-related potassium-1 (TREK-1) channels in the eEF2K-inhibition mediated potentiation of synaptic transmission. These findings reveal a novel pathway of eEF2K mediated regulation of hippocampal synaptic transmission. Further research is required to study whether such compounds could be beneficial for the development of mood disorder treatments with a fast-acting antidepressant response.

## Introduction

Neuronal network activity alteration can be encoded by regulation of a large population of synapses, which is known as an important mechanism for the determination of the resting state of brain activity and for balancing learning-mediated changes in synaptic transmission. Impaired resting state of brain activity underlies several diseases, such as, autism-spectrum disorders, bipolar disorder (Terwindt et al., 1998), schizophrenia (Dickman and Davis, 2009), as well as major depression disorder (Cross-Disorder Group of the Psychiatric Genomics Consortium, 2013).

Major depression disorder is a common but devastating mood disorder affecting many people. Studies suggest that inhibition of eEF2K plays an important role in the fast-acting antidepressant response of some of the available treatments (Autry et al., 2011; Zanos et al., 2016).

It was shown that a NMDA receptor blockade by ketamine led to an inhibition of eEF2K followed by enhanced protein synthesis and potentiation of hippocampal synapses that might mediate the antidepressant response of this compound (Autry et al., 2011; Duman et al., 2012; Dwyer and Duman, 2013; Monteggia et al., 2013). In addition, Zanos et al. (2016) stated that ketamine is metabolized *in vivo* to a ketamine derivative that shows antidepressant responses without blockage of NMDA receptors. The antidepressant effect of this derivative was still accompanied by a decrease in the phosphorylation of eEF2, an increase of synaptic transmission and neuronal network synchrony (Malinow, 2016; Zanos et al., 2016). eEF2K, also known as CaMKIII, belongs to the atypical alpha-kinase family (Ryazanov et al., 1997; Middelbeek et al., 2010) and one of its substrate – the eEF2 – has been linked to the regulation of protein synthesis (Taha et al., 2013), but also other substrates of eEF2K has been identified with potentially different outcome (Newman et al., 2013; Hu et al., 2014). The eEF2K itself underlies a complex dependency by upstream signaling pathways that results to a differently regulated eEF2K under various conditions and neuronal preparations (Kenney et al., 2014). It remains, however, unknown whether a specific eEF2K inhibition without modulation of up-stream or other signaling pathways is sufficient to alter synaptic transmission. To this end, we aimed to study the effects of direct eEF2K inhibition of hippocampal synaptic transmission and neuronal network activity in hippocampal slices and cultures.

Here, we used the selective and potent inhibitor A-484954 (Chen et al., 2011) and found that the inhibition of eEF2K caused an enhancement of synaptic transmission in the stratum radiatum of the hippocampal CA1 region that was independent of *de novo* protein synthesis and relied on p38 mitogen-activated protein kinase (MAPK) activity. We provided also evidence suggesting a presynaptic origin of the effect due to modulation of the vesicle release probability. As a potential target, we identified a barium-sensitive potassium channel, TREK-1. In addition, application of the eEF2K inhibitor increased the synchronization of neuronal network activity. These findings suggested a novel role of eEF2K in regulation of synaptic transmission under participation of p38 MAPK signaling and TREK-1 channels.

## Results

### Inhibition of eEF2K by A-484954 Elicits a Fast fEPSP Potentiation

In the search for specific inhibitors of eEF2K, also known as CaMKIII (Ryazanov et al., 1997; Middelbeek et al., 2010) the small molecule inhibitor A-484954 was identified from an Abbott compound library using high throughput screening (Chen et al., 2011). This compound possesses a half-maximal inhibitory concentration (IC50) against eEF2K of 0.28 μM.

**FIGURE 1:**
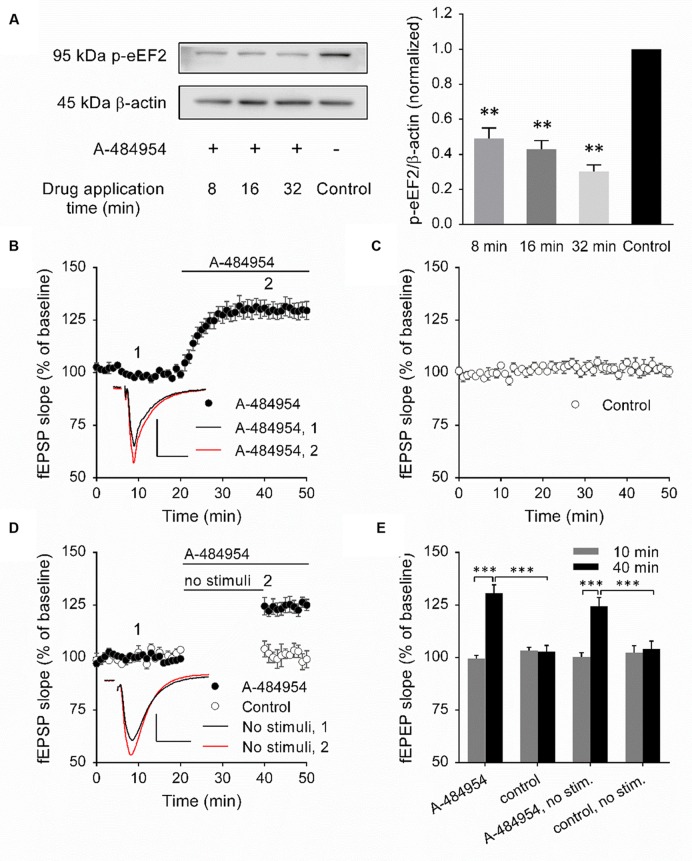
**Inhibition of eEF2K by A-484954 mediates an input nonspecific potentiation of synaptic transmission.** **(A)** The western blots indicate the decrease in eEF2 phosphorylation in response to the inhibition of eEF2K in comparison with drug-free samples. The bar graph summarizes the normalized phosphorylation level of eEF2 for drug applications of 8, 16, and 32 min. The application of A-484954 significantly prevented the phosphorylation of the eEF2K substrate eEF2. **(B)** Inhibition of eEF2K by 5 μM A-484954 (black circles, *n* = 9) elicited a potentiation of fEPSPs that reached a maximum within 10 min. **(C)** fEPSPs were recorded under drug-free conditions (control, white circles, *n* = 9). **(D)** The effect of A-484954 on synaptic transmission did not require evoked stimulation of the synapses. The insets indicate representative fEPSP traces before (black line) and after (red line) drug application. Scale bar, 0.5 mV/10 ms. **(E)** The bar graph summarizes the fEPSP slope values under different conditions and before (10 min) and during (40 min) drug application. The brackets and/or asterisks indicate a significant difference (^∗∗^*p* < 0.01; ^∗∗∗^*p* < 0.001) between control and drug groups. Horizontal black lines indicate the time of drug application.

To validate the inhibitory effect of A-484954 on eEF2K, we performed a biochemical protein analysis of the eEF2K substrate by eEF2 phosphorylation. We did not determine the total amount of eEF2 because the time between drug application and protein phosphorylation analysis was short and significant *de novo* protein synthesis or degradation of eEF2 was unlikely to have taken place. To this end, 5 μM A-484954 was applied to the eEF2K substrate for 8, 16, or 32 min, followed by snap freezing and storage at -80°C. On the day of analysis, the CA1 region was isolated and the resulting eEF2 phosphorylation level was examined (Yuanxiang et al., 2014). The western blots indicated that A-484954 significantly prevented the phosphorylation of eEF2 (**Figure 1A**). After verification of the efficient inhibition of eEF2K by A-484954, we looked into the effects of eEF2K inhibition on hippocampal synaptic transmission. We observed that the inhibition of eEF2K by A-484954 (5 μM) 20 min after stable baseline recordings resulted in a fast potentiation of fEPSPs (131 ± 3.8% at 40 min; *n* = 9) (**Figure 1B**), which differed significantly from drug-free experiments from 20 min onward. The fEPSP values were 105 ± 2.0% at 40 min (*n* = 9; **Figure 1C**).

### A-484954 Induced Potentiation Does Not Require an Evoked Synaptic Transmission

To determine whether the potentiation mediated by eEF2K inhibition required evoked synaptic activity, we took a fEPSP recording without stimulation of the fEPSPs during the initial 20 min of drug application (**Figure 1D**). Under these conditions, we observed a significantly different (*p* < 0.01) fEPSP potentiation (125 ± 3.6% at 45 min; *n* = 4) in comparison with drug-free experiments (control: 102 ± 2.3% at 45 min; *n* = 4). Because the effect of A-484954 on synapses was independent of evoked synaptic transmission, all synaptic inputs within the stratum radiatum were probably changed.

### Presynaptic Origin of the A-484954 Induced Synaptic Potentiation

**FIGURE 2:**
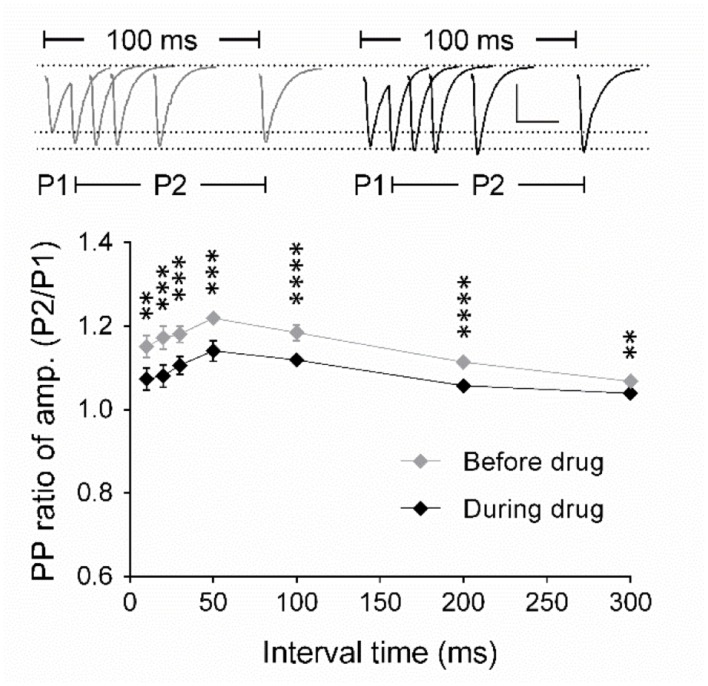
**Analysis of the paired-pulse ratio (PP ratio) of fEPSPs indicates a presynaptic origin of eEF2K-inhibition mediated potentiation.** The graph summarizes the PP ratio of fEPSP amplitudes for before (gray diamond, *n* = 9) and after (black diamond, *n* = 9) A-484954. PP ratios after drug application differed significantly for all inter-stimulus intervals (bracket, ^∗∗^*p* < 0.01, ^∗∗∗^*p* < 0.001, ^∗∗∗∗^*p* < 0.0001, paired *t*-test). Insets show representative fEPSP traces before and after drug application. Scale bar: 0.5 mV/20 ms.

**FIGURE 3:**
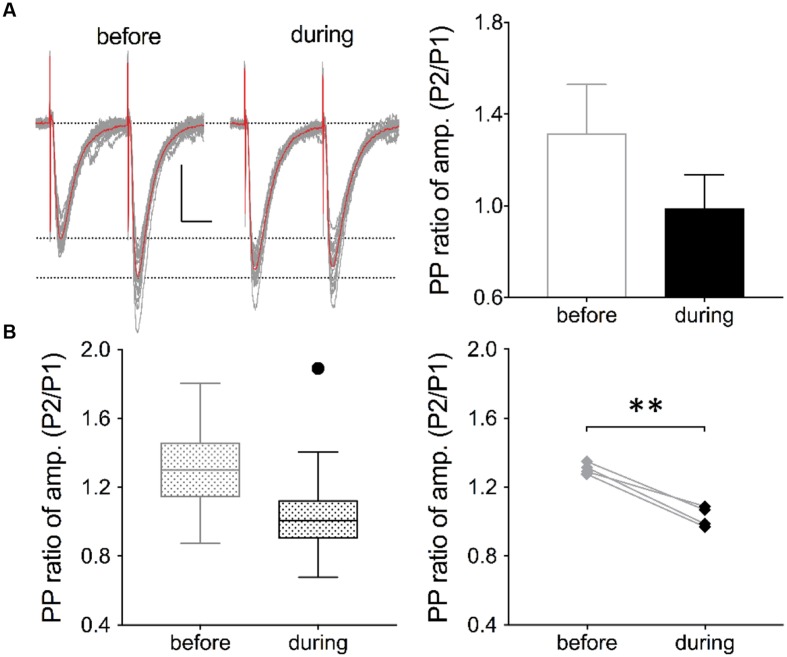
**Inhibition of eEF2K alters the PP ratio of evoked EPSCs.** **(A)** Traces of 19 evoked EPSCs (gray lines) before and after drug application are shown. The interstimulus interval was 50 ms. The traces of averaged EPSCs are shown in red. Scale bar: 50 pA/20 ms. Right: Analysis of the traces (19 EPSCs) revealed that the averaged PP ratio value was reduced after 10 min of drug application (black circle) in comparison with the baseline value (gray circle). **(B)** The left graph summarizes the distribution of PP ratios before and during drug application of all recordings in four neurons. The black dot represents an outlier. To the right, the averaged values of four experiments are presented and the significant effect has been indicated with a bracket and ^∗∗^ (*p* < 0.01, *p* = 0.0027).

**FIGURE 4:**
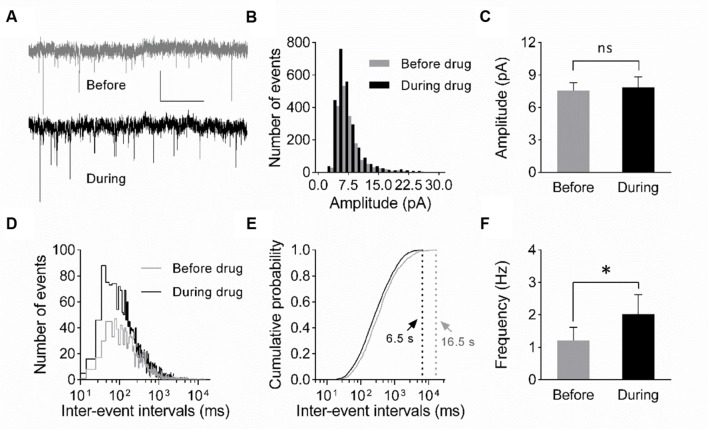
**Increase in mEPSC frequency suggests a presynaptic origin of eEF2K-inhibition mediated potentiation.** **(A)** Representative traces of whole-cell voltage-clamp recordings of mEPSCs before (gray) and during (black) drug application are depicted. Scale bar: 10 pA/2 s. **(B)** The histogram summarizes the distribution of mEPSC amplitudes before (gray bars) and during (black bars) drug application over all events of all recordings (*n* = 7). **(C)** The mean amplitude before (gray bar) and during (black bar) drug application do not differ significantly (*n* = 7; 7.6 ± 0.7 pA versus 7.8 ± 1.0 pA; ns: *p* = 0.94). **(D)** The histogram indicates the distribution of the inter-event intervals for before (gray) and during (black) drug application for all events of all neurons analyzed (*n* = 7). **(E)** The cumulative probability of inter-event intervals of mEPSCs before (gray) and during (black) drug application differs. Vertical dashed lines indicate the maximal values of the inter-event intervals before (gray) and during (black) drug application. **(F)** The mean mEPSC frequencies differ significantly (^∗^*p* = 0.0148) before and during drug application (*n* = 7, 1.2 ± 0.4 Hz versus 2.0 ± 0.6 Hz).

To identify the origin of the effect of the drug on synaptic transmission, we analyzed the paired-pulse (PP) ratio of fEPSPs and the frequency and amplitude of miniature excitatory postsynaptic currents (mEPSCs). Using field potential recordings of synaptic transmission, we detected that the PP ratio of fEPSPs decreased significantly after drug application for all inter-stimulus intervals tested in comparison with PP ratios before drug application (*p* < 0.05; **Figure 2**). We concluded that these data indicated a modulation of the presynaptic release probability, which we aimed to investigate in detail using whole-cell voltage clamp analysis of evoked EPSCs and mEPSCs (**Figures 3** and **4**).

To verify whether a presynaptic origin of the potentiation mediated by eEF2K inhibition underlies the potentiation of synapses, we performed whole-cell voltage clamp recordings of evoked EPSCs using a paired pulse stimulation protocol (interstimulus interval: 50 ms) in hippocampal CA1 neurons. The recordings revealed that the application of A-484954 significantly reduced the paired pulse ratio of evoked EPSCs (**Figure 3**). We also performed whole-cell voltage clamp recordings of mEPSCs in CA1 pyramidal cells. Tetrodotoxin (1 μM) and bicuculline (10 μM) were added to prevent action potential-mediated glutamate release and to block inhibitory postsynaptic currents, respectively. The recordings revealed that the application of A-484954 evoked an increase in mEPSC events over time (**Figures 4A,B**) with no alteration of their amplitudes (**Figure 4C**) in comparison with drug-free conditions. Thus, inter-event intervals became smaller (**Figure 4D**), and the maximal inter-event intervals for before and during drug application shifted from 16.5 to 6.5 s (**Figure 4E**) respectively. The significantly higher probability of mEPSC appearance after drug application was also represented by an increase in mEPSC frequency in comparison with the value before drug application (**Figure 4F**).

### eEF2K-Inhibition Mediated Potentiation Does Not Rely on Protein Synthesis

**FIGURE 5:**
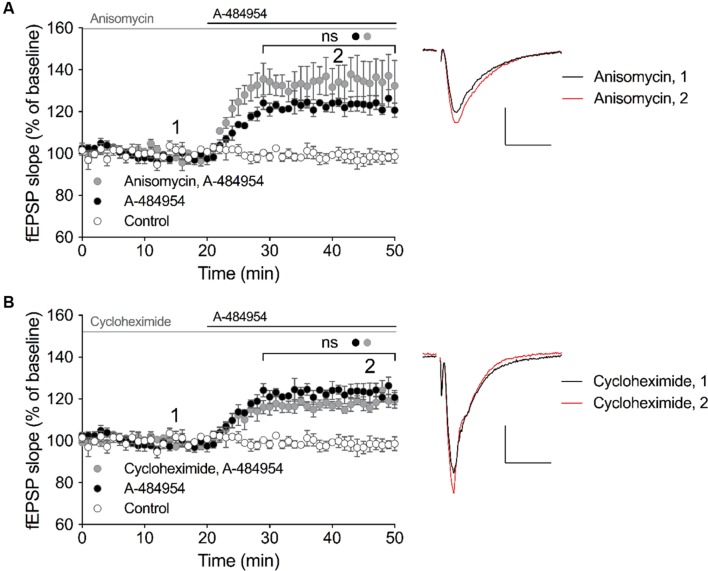
**eEF2K-inhibition mediated-fEPSP potentiation does not rely on protein synthesis.** **(A)** The graph summarizes the mean values of normalized fEPSP slopes for different time points and experimental conditions (control: white circles, *n* = 4; 5 μM A-484954: black circles, *n* = 4; 20 μM anisomycin: gray circles, *n* = 4). **(B)** Cycloheximide (gray circles, 100 μM, *n* = 4) did not alter the A-481954-mediated (black circles, *n* = 4) potentiation of fEPSP. The potentiation of fEPSP remained significantly higher than that observed under control conditions (no A-481954, white circles, *n* = 4). Horizontal black lines indicate the application period of A-484954 and gray lines indicates the application time of protein synthesis inhibitors. The brackets enclose time points with non-significant (ns) differences between conditions. To the right of the graphs, representative fEPSP traces for the time points 1 and 2 are depicted. Scale bar: 0.5 mV/10 ms.

eEF2K and one of its substrate – the eEF2 – has been linked to the regulation of protein synthesis, however, also other substrates of eEF2K has been identified that might promote protein-synthesis independent regulation of cellular processes. Thus, we were interested to determine whether the effect of A-494854 on synaptic transmission was dependent on protein synthesis. To this end, we pre-incubated acute hippocampal slices with the protein synthesis inhibitor anisomycin (20 μM) for 1 h before the baseline recording. After an additional 20-min baseline recording in the presence of the inhibitor, A-484954 (5 μM) was applied to the artificial cerebrospinal fluid causing a fast potentiation of fEPSP. No significant difference (*p* = 0.47) was found between the A-484954-treated group (119 ± 1.5% at 40 min; *n* = 4; **Figure 5A**) and pretreated group (131 ± 8.8% at 40 min; *n* = 4; **Figure 5A**), indicating that anisomycin did not prevent eEF2K-inhibition mediated fEPSP potentiation.

To verify the previous data, we pretreated hippocampal slices with a different protein synthesis inhibitor, cycloheximide (100 μM), for 1 h. After 20 min of a stable baseline recording, A-484954 (5 μM) was applied and induced a fast potentiation of fEPSP. No significant difference (*p* = 0.18) was found between the A-484954-treated group (119 ± 1.5% at 40 min; *n* = 4; **Figure 5B**) and pretreated group (117 ± 2.1% at 40 min; *n* = 4; **Figure 5B**), indicating that cycloheximide did not prevent eEF2K-inhibition mediated fEPSP potentiation. These experiments provided evidence that eEF2K-inhibition mediated potentiation does not rely on protein synthesis.

### eEF2K-Inhibition Mediated fEPSP-Potentiation Does Not rely on Ca^2+^/Calmodulin-Dependent Protein Kinase II (CaMKII), Protein Kinase C (PKC) or Protein Kinase A (PKA)

**FIGURE 6:**
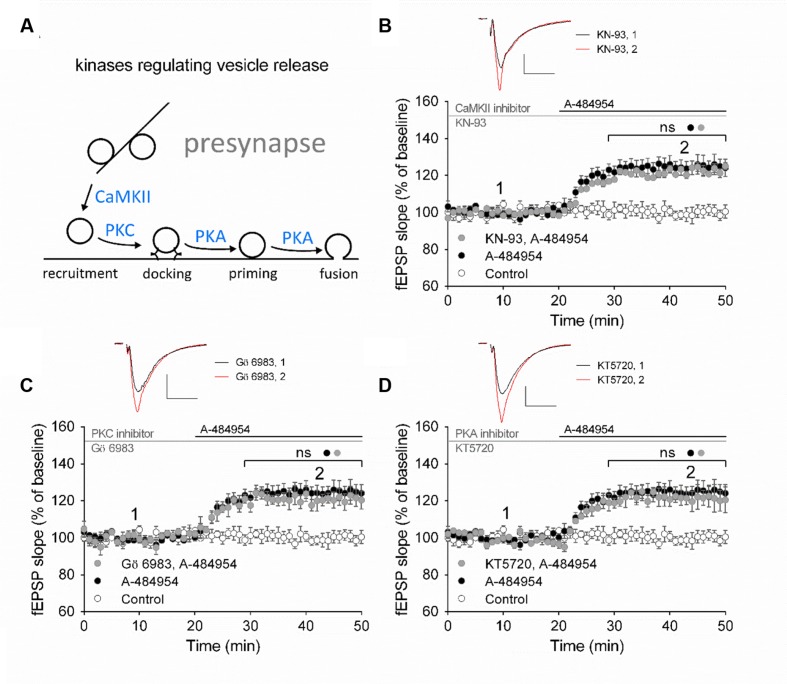
**eEF2K-inhibition mediated fEPSP-potentiation does not rely on CaMKII, PKC or PKA.** **(A)** The schemata outlines the relation of kinases in the regulation of vesicle release. **(B)** Inhibition of CaMKII by KN-93 does not attenuate drug-induced potentiation (control: white circles = 6; 5 μM A-484954: black circles, *n* = 6; 20 μM KN-93: gray circles, *n* = 4). **(C)** The graph represents data of PKC inhibition experiments (gray circles: *n* = 4) in comparison to drug-free (control: white circles, *n* = 6) and A-484954 (black circles: *n* = 6) experiments. **(D)** Inhibition of PKA (KT5720: gray circles, *n* = 4) did not alter the time course of fEPSPs in comparison with experiments with the application of A-484954 alone (black circles, *n* = 6). The drug-induced potentiation remained significantly different from that in control experiments (white circles, *n* = 6). Black and gray horizontal lines represent the application period of A-484954 and inhibitors, respectively. Brackets enclose periods with significant differences (ns: *p* > 0.05) between groups as specified by the circles. fEPSP traces at time points 1 and 2 are depicted. Scale bars: 0.5 mV/10 ms.

Presynaptically localized CaMKII, PKC, and PKA have been reported to be involved in the regulation of vesicle release probabilities (Abeliovich et al., 1993; Mayford et al., 1996; Patterson et al., 2001). A simplified schemata of potential interactions is shown in **Figure 6A** (modified from Leenders and Sheng, 2005). To investigate the potential involvement of these key pathways we perfused acute hippocampal slices with specific inhibitors for 1 h before A-484954 application. We did not observe any change in the potentiation of fEPSP mediated by eEF2K inhibition by specific inhibitors of CaMKII, PKC, or PKA (**Figure 6**). Specifically, the CaMKII inhibitor KN-93 (20 μM) did not attenuate A-484954 induced fEPSP potentiation (**Figure 6B**); the PKC inhibitor Gö 6983 (Simons et al., 2012; Vasefi et al., 2013) did not alter the dynamic of the A-484954 mediated effect on fEPSPs (**Figure 6C**); and the inhibition of PKA by KT5720 did not result in any modulation of the drug-induced synaptic potentiation (**Figure 6D**).

### eEF2K-Inhibition Mediated fEPSP-Potentiation Relies Partially on Intracellular Calcium Release

**FIGURE 7:**
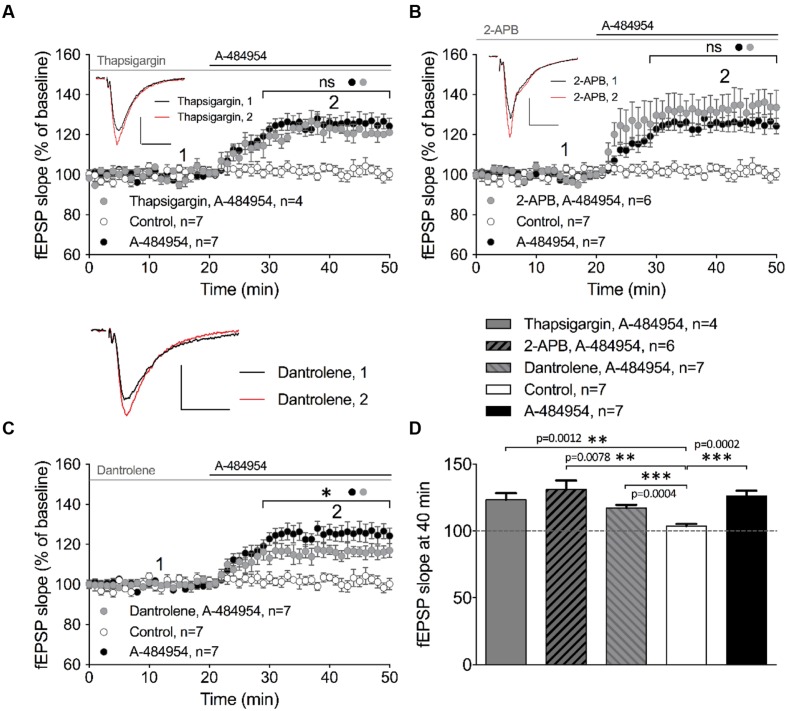
**eEF2K-inhibition mediated fEPSP-potentiation relies partially on intracellular calcium release.** **(A)** Application of thapsigargin (10 μM thapsigargin: gray circles, *n* = 4) did not alter A-484954-induced fEPSP potentiation over time (drug-free: control, white circles, *n* = 7; 5 μM A-484954 alone; black circles, *n* = 7). **(B)** Inhibition of InsP3R-mediated intracellular calcium release (100 μM 2-APB: gray circles, *n* = 6) did not alter the slope of fEPSP potentiation (A-484954: black circles, *n* = 7), which remained significantly different from that in drug-free experiments (white circles, *n* = 7). **(C)** Inhibition of ryanodine receptors (20 μM dantrolene: gray circles, *n* = 7) significantly attenuated (*p* = 0.037) the potentiation of fEPSP mediated by eEF2K inhibition in comparison with A-484954 alone (black circles, *n* = 7). However, experiments with dantrolene gave significantly elevated results in comparison with drug-free measurements (control: white circles, *n* = 7). Black and gray lines indicate A-484954 and inhibitor application times, respectively. Brackets indicate significance levels (^∗^*p* < 0.05; ns: *p* > 0.05) between groups (circles). fEPSP traces for the time points 1 and 2 are depicted. Scale bars: 0.5 mV/10 ms. **(D)** The bar graph summarizes the observations and significance levels (brackets, ^∗∗^*p* < 0.01, ^∗∗∗^*p* < 0.001) for the different experimental conditions in comparison with drug-free (control) experiments for fEPSP slope values at the 40-min time point.

Intracellular calcium release from endoplasmic reticulum (ER) has been reported to play an important role in the regulation of neural properties, such as excitability of hippocampal pyramidal cells (Borde et al., 2000; Shah and Haylett, 2000; Shirasaki et al., 2001), frequency and amplitude of mEPSCs (Sharma and Vijayaraghavan, 2003), and presynaptic plasticity (Emptage et al., 2001; Liang et al., 2002; Martin and Buno, 2003). We therefore investigated whether the potentiation of fEPSP mediated by eEF2K inhibition requires calcium release from intracellular calcium stores. Firstly, we tested thapsigargin, a non-competitive inhibitor of sarco-endoplasmic reticulum Ca^2+^-ATPases (SERCA) that causes depletion of intracellular calcium stores. We observed that 10 μM thapsigargin provoked an enhancement of basal synaptic transmission that stabilized within 1 h of incubation (data not shown). After a 20-min stable baseline recording under thapsigargin, application of A-484954 (5 μM) enhanced the fEPSP slopes (121 ± 4.8% at 40 min; *n* = 4; **Figure 7A**). The increase in fEPSP slopes did not differ from the potentiation induced by A-484954 alone (126 ± 3.9% at 40 min; *n* = 7; **Figure 7A**), but was significantly larger than that observed in drug free experiments (**Figure 7D**).

In a second step, we evaluated the involvement of the inositol trisphosphate receptor (InsP3R) in the drug-induced potentiation. The intracellular calcium release activated by InsP3R plays important roles in proliferation, development, learning, and memory (Bosanac et al., 2002). We tested the InsP3R antagonist aminoethoxydiphenyl borate (2-APB) and observed that pretreatment and co-application with A-484954 did not affect the potentiation of fEPSPs. The co-application of A-484954 with an InsP3R blocker induced a fast fEPSP potentiation of 132 ± 7.1% at 40 min (*n* = 6; **Figure 7B**). This potentiation was similar to that observed with the A-484954 alone (126 ± 3.9% at 40 min; *n* = 7; **Figure 7B**) and was significantly larger than baseline values in drug-free experiments (104 ± 1.7% at 40 min; *n* = 7; **Figure 7D**). Therefore, the data suggest that calcium release from InsP3R dependent calcium stores is not involved in the eEF2K-inhibition mediated effect on synaptic potentiation.

Ryanodine receptors (RyRs) at the ER are sensitive to caffeine and ryanodine and are linked to thapsigargin non-sensitive calcium stores. These channels respond to minor variations in intracellular calcium concentrations that causes further release of calcium (Zucchi and Ronca-Testoni, 1997). Blockage of RyRs by dantrolene (20 μM) prevented A-484954 (5 μM) induced fEPSP potentiation to some degree. Co-application of dantrolene and A-484954 resulted in a potentiation of fEPSP of 117 ± 2.2% at 40 min (*n* = 7; **Figure 7C**) in comparison with A-484954 alone (126 ± 3.9% at 40 min, *n* = 7; **Figure 7C**). The remaining fEPSP potentiation was still significantly larger than the fEPSP values of drug free experiments (**Figure 7D**). Thus, only the calcium release mediated by the activation of RyRs played a partial role in eEF2K-inhibition mediated potentiation.

### Inhibition of p38 MAPK Attenuates eEF2K-Inhibition Mediated fEPSP-Potentiation

**FIGURE 8:**
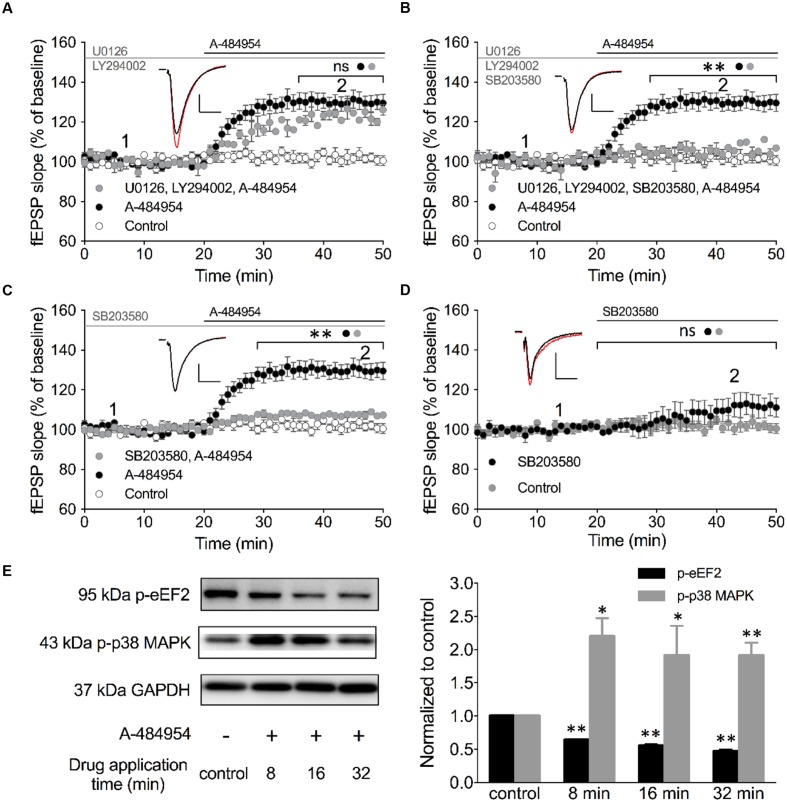
**Inhibition of p38 MAPK attenuates eEF2K-inhibition mediated fEPSP-potentiation.** **(A)** The line graph summarizes the resulting fEPSP slopes over time under three experimental conditions (control: white circles; 5 μM A-484954: black circles; 10 μM U0126 plus 10 μM LY492002: gray circles). U0126 and LY492002 are inhibitors of MEK 1/2 and PI3K, respectively. **(B)** Additional application of a p38 MAPK inhibitor (10 μM SB203580) to the MEK 1/2-PI3K inhibitor cocktail (gray circles) significantly attenuated the A-484954-mediated potentiation of fEPSP (black circles; *p* < 0.01, *p* = 0.0024 at 40 min). The resulting fEPSPs were similar in size in comparison with drug-free experiments. **(C)** Application of the p38 MAPK inhibitor alone (gray circles) also prevented the A-484954-mediated potentiation of fEPSP (black circles) significantly (*p* < 0.01, *p* = 0.0003 at 40 min). **(D)** Application of the p38 MAPK inhibitor alone (black circles) did not alter the fEPSP baseline (*p* = 0.07 at 45 min) in comparison with drug-free baseline values (gray circles). Brackets with asterisks indicate the significance level (^∗∗^*p* < 0.01 or ns: *p* > 0.05) between the groups specified by circles. fEPSP traces at the time points 1 (black) and 2 (red) are depicted. Scale bars: 0.5 mV/10 ms. **(E)** The left panel illustrates representative Western blots for p-eEF2, p-p38 MAPK (Thr180/Tyr182) and GAPDH in total protein lysates from the CA1 region without (-) and after 5 μM A-484954 treatment for 8, 16, and 32 min (+). The bar graph summarizes normalized western blot data for p-EF2 and p-p38 MAPK. The phosphorylation levels of p38 MAPK (Thr180/Tyr182, gray bar) and eEF2 (black bar) were significantly different from the control (drug-free) measurements (^∗∗^*p* < 0.01; *n* = 3/group).

We looked into various other signaling pathways also known to be involved in the regulation of vesicle release and conducted experiments using specific inhibitors against PI3K-Akt and MEK/ERK (English and Cobb, 2002). LY294002 is an inhibitor of PI3K that has been shown to be effective at a concentration of 10 μM in acute hippocampal slices (Chang et al., 2014). In addition, we tested U0126 as an inhibitor of MEK 1/2 at a concentration of 10 μM that also has been shown to be effective in hippocampal slices (Kidambi et al., 2010). Pretreatment of hippocampal slices with a cocktail of LY294002 and U0126 for 1 h did not alter fEPSP potentiation induced by A-484954. The resulting fEPSP potentiation was 123 ± 2.0% (*n* = 4) at 40 min (**Figure 8A**) that did not differ (*p* = 0.24) from the fEPSP values of the A-484954 group (131 ± 4.2%, *n* = 9), but was still significantly larger than that in the control group (drug free; 102 ± 2.4%, *n* = 9).

p38 MAPKs are stress-activated protein kinases that are activated through extracellular stress, cytokines, growth factor, and osmotic shock (Cuenda and Rousseau, 2007). In addition, the p38 MAPK pathway plays an important role in synaptic plasticity and neurodegenerative diseases (Correa and Eales, 2012). The p38 MAPK family comprises four isoforms: p38α, p38β, p38γ and p38δ that act on a variety of substrates, e.g., microtubule-associated protein tau and transcription factors. SB203580 is a highly specific cell-permeable inhibitor (English and Cobb, 2002) of p38α and p38β that can inhibit these isoforms at 10 μM in hippocampal slices (Capron et al., 2006; Hasegawa et al., 2014). We tested the involvement of p38 MAPK in the eEF2K-inhibition mediated fEPSP potentiation by adding 10 μM SB203580 to the Akt/MEK 1/2 inhibitor cocktail (**Figure 8B**). We noticed that this cocktail efficiently eliminated the previously robust A-484954 induced fEPSP potentiation. The fEPSPs (105 ± 4.2% at 40 min; *n* = 4; **Figure 8B**) differed significantly (*p* = 0.0024) for all time points tested in comparison with A-494854 alone experiments (131 ± 3.8% at 40 min; *n* = 9; **Figure 8B**). These data suggested that p38 MAPK is required for the eEF2K-inhibition mediated fEPSP potentiation.

To confirm the role of p38 MAPK, we incubated the acute hippocampal slices for 1 h in 10 μM SB203580 containing ACSF (**Figure 8C**). Consistent with our previous result, 10 μM SB203580 alone was sufficient to attenuate the A-484954 induced fEPSP potentiation, with a significant difference (*p* = 0.0003) between the pretreated group (107 ± 0.4% at 40 min; *n* = 6; **Figure 8C**, gray circle) and the non-pretreated group (131 ± 3.8% at 40 min; *n* = 9; **Figure 8C**, black circle). Application of SB203580 alone did not show a significant (*p* = 0.07) effect on the fEPSP baseline (113 ± 5.6% at 45 min; *n* = 8; **Figure 8D**, black circles). To further determine whether the inhibition of eEF2K results in an increase of p38 MAPK phosphorylation, we collected slices after 8-, 16-, or 32-min applications A-484954 (5 μM) for whole-protein biochemical analysis. The western blot data indicated that A-484954 (5 μM) induced a rapid phosphorylation of p38 MAPK (**Figure 8E**), suggesting that p38 MAPK phosphorylation is altered in response to eEF2K inhibition and that the p38 MAPK takes part in the A-484954 induced enhancement of synaptic transmission.

### Barium Sensitive Potassium Channels Are Involved in eEF2K-Inhibition Mediated fEPSP Potentiation

p38 MAPK has been reported to regulate hyperpolarization-activated cyclic nucleotide-gated (HCN) channels in hippocampal pyramidal neurons (Poolos et al., 2006; Jung et al., 2010). We tested whether HCN channels were involved in eEF2K-inhibition mediated fEPSP potentiation. In these experiments, the blocker (ZD7288 or Zatebradine) was applied from the start of baseline recording to the end of the experiment and showing no effect on fEPSP potentiation (data not shown).

**FIGURE 9:**
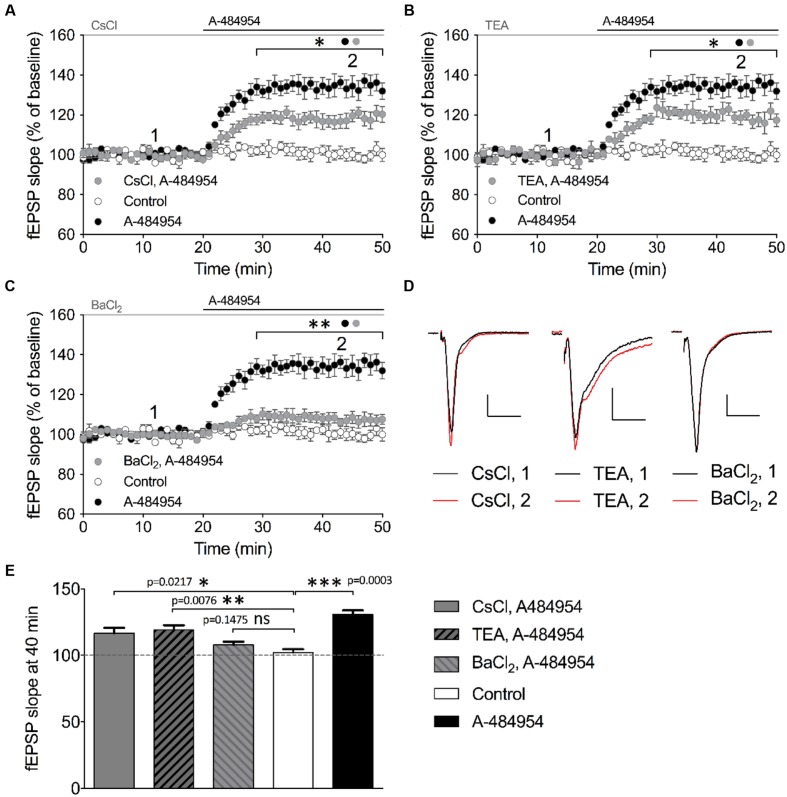
**Barium sensitive potassium channels are required for eEF2K-inhibition mediated fEPSP potentiation.** **(A)** The graph indicates the fEPSP slopes over time under three experimental conditions (control: white circles, *n* = 4; 5 μM A-484954: black circles, *n* = 4; 2 mM CsCl_2_: gray circles, *n* = 4). **(B)** Blockage of TEA (10 mM, gray circles)-sensitive potassium channels partially but significantly attenuated A-484954-mediated fEPSP potentiation (black circles, *n* = 4). The remaining fEPSP potentiation was still significantly different from that of drug-free experiments (white circles, *n* = 4). **(C)** Pre-application with 400 μM BaCl_2_ significantly prevented A-484954-mediated fEPSP potentiation (*p* < 0.01). fEPSP slopes were similar in size to those with drug-free experiments (control: white circles, *n* = 4). Horizontal black and gray lines indicate A-484954 and, CsCl, TEA, or BaCl_2_ application times, respectively. Brackets enclose the time points of significant differences (^∗^*p* < 0.05; ^∗∗^*p* < 0.01) between groups (circle) per time point. **(D)** Representative fEPSP traces for the time points 1 and 2 and compounds are depicted. Scale bars: 0.5 mV/10 ms. **(E)** The bar graph summarizes the observations and significance levels (brackets; ^∗^*p* < 0.05; ^∗∗^*p* < 0.01; ^∗∗∗^*p* < 0.001) for different experimental conditions in comparison with drug-free (control: white bar) experiments for fEPSP slope values at the 40-min time point.

We then tested the broad-spectrum potassium channel blocker cesium chloride (CsCl). After a stable baseline recording with CsCl (2 mM), we washed in A-484954 (5 μM) and observed a fast potentiation of fEPSP (117 ± 4.0% at 40 min; *n* = 4; **Figure 9A**) that was significantly smaller (*p* = 0.031) than the values observed in the A-484954 group (135 ± 4.2% at 40 min; *n* = 4; **Figure 9A**). Likewise, we tested the effect of 10 mM TEA (**Figure 9B**) and 400 μM BaCl_2_ (**Figure 9C**) on eEF2K-inhibition mediated potentiation. These experiments indicated that TEA only partially (*p* = 0.046) affected the drug induced fEPSP potentiation reaching 119 ± 3.5% (TEA, 40 min, *n* = 4). However, similar experiments with BaCl_2_ attenuated the drug induced fEPSP potentiation fully as indicated by the fEPSP slope value of 107 ± 2.3% at the 40 min (*n* = 6, *p* = 0.0003). Statistical analysis revealed a significant difference for all blockers tested in comparison with experiments with the drug alone. However, the resulting potentiation for TEA and CsCl, but not for BaCl_2_, remained significantly different in comparison with the drug-free experiments (**Figures 9D,E**). These results suggest that the eEF2K inhibition mediated fEPSP potentiation requires especially the contribution of barium sensitive potassium channels that are not affected by TEA or CsCl (Wulff and Zhorov, 2008).

### The Outward Rectifier Potassium Channel TREK-1 Is Involved in eEF2K-Inhibition Mediated fEPSP Potentiation

**FIGURE 10:**
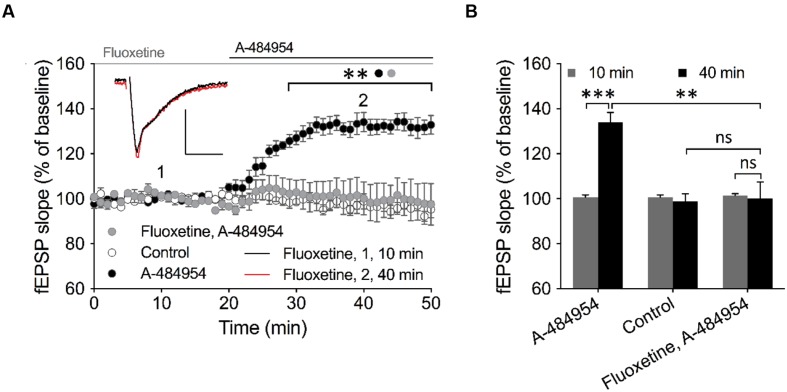
**TREK-1 is involved in eEF2K-inhibition mediated fEPSP potentiation.** **(A)** The graph outlines fEPSP-slopes over time under three experimental conditions (control: white circles, *n* = 4; 5 μM A-484954: black circles, *n* = 6; 100 μM Fluoxetine + 5 μM A-484954: gray circles, *n* = 6). The pretreatment and co-application of hippocampal slices with Fluoxetine and A-484954 significantly blocked A-484954 mediated fEPSP potentiation (*p* < 0.01). The resulting fEPSP slopes were similar in size as in the non-drug experiments (control: white circles). Horizontal black and gray lines indicate the A-484954 and Fluoxetine application period respectively. fEPSP traces for fluoxetine co-application experiments are shown. Scale bars: 0.5 mV/10 ms. **(B)** The bar graph summarizes averaged fEPSP-slope values for different experiments at the 10- and 40-min time points. Brackets enclose experimental groups with significant difference (^∗∗^*p* < 0.01, *p* = 0.0024; ^∗∗∗^*p* < 0.001, *p* = 0.0003).

Insight into barium sensitive potassium channel family (Wulff and Zhorov, 2008) gave us a candidate – the outward-rectifier background potassium channel TREK-1 (also named KCNK2), which is involved in the control of resting membrane potential (Ma et al., 2011). This channel has been previously linked to play important roles in neuroprotection against epileptic seizures and ischemia. To study a potential involvement of TREK-1 in the effects of A-484954 on hippocampal synaptic transmission, we investigated whether fluoxetine, which has been proven to block TREK-1 (Kennard et al., 2005; Eckert et al., 2011; Moha ou Maati et al., 2011; Sandoz et al., 2011; Xi et al., 2011) in a voltage-independent manner (Honore et al., 2002; Kennard et al., 2005) and also interferes with other signaling pathways (Edgar et al., 1999; Fumagalli et al., 2005; Stepulak et al., 2008), attenuates the potentiation of synapses. This compound has been widely prescribed as a fast-acting treatment of depression disorders, however, with psychotropic side effects (Alboni et al., 2015; Bastos et al., 2015; Liang et al., 2015). We observed that pre-incubation and co-application of fluoxetine with A-484954 prevented the potentiation of fEPSP-slopes (**Figures 10A,B**).

### Inhibition of eEF2K Synchronizes Activity of Neurons in Primary Hippocampal Cell Cultures

**FIGURE 11:**
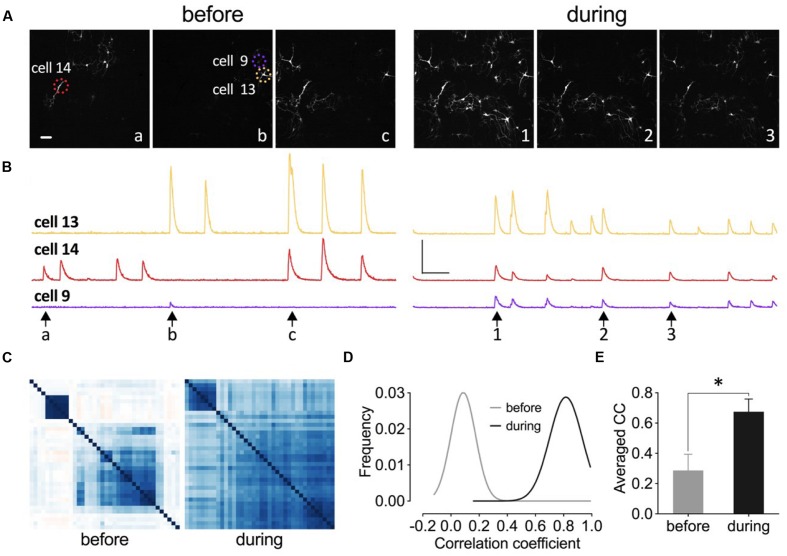
**Inhibition of eEF2K synchronizes neuronal activity pattern in primary hippocampal cell cultures.** **(A–C)** Images, traces, and heat maps in the figure represent data from one representative experiment before and during drug application. **(A)** The time points for the representative frames of a fluorescence image sequence are indicated under the traces in **(B)** with arrows and letters or numbers. Dashed circles indicate representative neurons (14, 9, and 13). Scale bar, 100 μm. **(B)** The time courses of changes in fluorescence intensity for three neurons (color-coded throughout the figure) before and during drug application are presented. Vertical scale bar: 2000 (F-F_0_/F_0_) and horizontal bar: 5 s. **(C)** The degree of synchronized spike activity of neurons to each other are represented by a Pearson’s correlation based hierarchical clustering matrices. **(D)** Theoretical Gaussian fits for the frequency distribution histograms of Pearson’s correlation coefficients were created for all neuronal pairs of three cultures analyzed before and during drug application. **(E)** The bar graph summarizes the mean values of correlation coefficient (CC) matrices. The brackets and asterisks indicate the degree of significant difference (^∗^*p* < 0.05) of the coefficients before (0.29 ± 0.11) and after drug application (0.67 ± 0.08).

Increased synchrony of neuronal network has been implicated as one of the mechanisms to counteract depression and it has been shown that a ketamine metabolite with anti-depressant effects increased Gamma oscillation *in vivo* (Zanos et al., 2016). Here we tested whether the application of this eEF2K inhibitor would enhance neuronal network synchronization. We transduced neurons of primary hippocampal cell cultures with the genetically encoded calcium sensor GCamp6f and analyzed the changes in spontaneous fluorescence intensity within individual neurons of the network (**Figure 11**). As it is indicated in the fluorescence images at different time points in **Figure 11A**, only a few neurons had a synchronized activity pattern under drug-free conditions (**Figure 11A**, left). The application of 5 μM A-484954 enhanced the number of synchronized neurons per frame (**Figure 11A**, right). The peaks of the fluorescence intensity traces of these three cells (circled in **Figure 11A**, left) appear simultaneously after drug application (**Figure 11B**), indicating the highest degree of synchronized activity pattern.

To quantify the degree of synchronization of the neuronal pairs within one culture dish, a Pearson’s correlation matrix was calculated and the individual coefficients were presented as a color-coded heat map matrix (**Figure 11C**). The coefficients are color-coded from dark red over white to dark blue, which correspond to -1, 0, and 1, respectively. This matrix illustrates that the degree of synchronization increased during drug application.

We conducted analyses of two more cell cultures and analyzed more than 130 neurons. The calculation of the Pearson’s correlation coefficients and construction of the Gaussian fit for the frequency distribution histograms (**Figure 11D**) revealed that A-484954 efficiently increased the coefficients and therefore the synchronicity of neuronal activity patterns in the cultures. In addition, a comparison of the averaged coefficients, for the three cultures, confirmed the significant difference between the results before and during drug application (**Figure 11E**). Thus, we conclude that A-484954 not only alters the efficiency of synaptic transmission, but also increases the level of synchronicity of neuronal activity pattern.

## Discussion

To investigate whether direct inhibition of eEF2K causes a potentiation of synaptic transmission, we employed a new small molecule eEF2K inhibitor to hippocampal slices and analyzed the effects on the synaptic transmission. The compound A-484954 was detected in an Abbott compound library using high throughput screening in a cell free enzymatic assay against eEF2K. The compound has an IC50 of 0.28 μM against eEF2K with little activity against a wide panel of kinases [see in Figure 3A the selective kinase profile of the Chen et al. (2011) publication]. In one of the initial papers, the authors tested the effect of A-484954 on eEF2K in comparison to siRNA assays. They could indicate the similarity of the effects of reduction in eEF2 phosphorylation but not growth of cancer cell. The widely used NH125, however, induced eEF2 phosphorylation by multiple pathways, instead of suppressing it. In our cell-based assay (acute hippocampal slice), we could show that the utilized concentration of A-484954 effectively inhibited the eEF2K activity by analyzing the phosphorylation level of eEF2. This was not a test for the compound’s specificity, because trials of a compound’s specificity in a cell-based system is hindered by multiple crosslinks between different signaling pathways inducing positive or negative feedback regulation of other key regulators. Thus, only cell free assays against a variety of targets would be an appropriate approach to test a compound’s specificity (Bain et al., 2007; Chen et al., 2011), thus we refer by the interpretation of our data on publications (Chen et al., 2011; Usui et al., 2015; Wang et al., 2015).

Using the eEF2K inhibitor A-484954, we showed that the inhibition of eEF2K enhances synaptic transmission by up-regulation of the vesicle release probability. In addition, the enhancement of synaptic transmission by A-484954 reached its maximum also without active stimulation of Schaffer-collateral fibers. This experiment showed that the enhancement of synaptic transmission by A-484954 did not require postsynaptic depolarization induced by an evoked glutamate release. Thus, it is likely that A-484954 is inducing a potentiation of most excitatory synapses in the stratum radiatum. Induction of synaptic potentiation by ketamine and MK-801 does also not rely on evoked synaptic transmission (Nosyreva et al., 2013). In addition, the potentiated synaptic response was accompanied by an enhanced release probability of vesicles without affecting the amplitude of synaptic events. This type of change has been commonly attributed to an alteration of the pre-synaptic glutamate release machinery. A modulation of the glutamate receptor density or conductance should have changed the EPSC amplitude without affecting the PP ratio of synaptic transmission. Thus, the presynaptic origin of the eEF2K effect is different from the ketamine and MK-801 induced synaptic potentiation by postsynaptic localized mechanisms (Nosyreva et al., 2013).

### Mechanisms of eEF2K-Inhibition Mediated Potentiation of Synaptic Transmission

Ketamine and MK-801 have been found to alter the postsynaptic AMPA receptor compensation under the involvement of mTOR, eEF2K and BDNF (Autry et al., 2011; Duman et al., 2012; Dwyer and Duman, 2013; Monteggia et al., 2013; Nosyreva et al., 2013). We like to note that the differences between our and ketamine related study is that the compound A-484954 is inhibiting eEF2K without direct activation of upstream signaling pathways through the interference with glutamate receptors. This resulted in a very fast synaptic response to the compound, while Ketamine and MK-801 related experiments require at least 30 min to alter synaptic transmission.

One other difference of the effect of direct eEF2K inhibition on synaptic responses in comparison with data from ketamine and MK-801 related experiments (Nosyreva et al., 2013) is its protein synthesis independency. We address this discrepancy to the complexity of interconnected signaling pathways when compounds have a large number of interaction partners. Ketamine itself is well known to affect a wide range of cellular processes by modulation, e.g., of cholinergic (Yamakura et al., 2000; Lydic and Baghdoyan, 2002), or aminergic system (dopamine and noradrenaline) (Kubota et al., 1999; Kamiyama et al., 2011; Wang et al., 2012; Sleigh et al., 2014) and for its derivative the targets are even unknown (Zanos et al., 2016). Thus, a distinct modulation of different signaling pathways might promote protein synthesis dependent modulation of synaptic transmission. Only proteomic or phospho-proteomic studies might be able to visualize the complexity of altered pathways, e.g., for MK-801 (Guest et al., 2015). In addition, many studies of the eEF2K function relied on the inhibitors NH125 and Rottlerin that have been shown recently to act also in an unpredicted fashion indicating the necessity of further studies to clarify the interaction partners (Davies et al., 2000; Bender et al., 2016). One of the identified eEF2K substrates (Newman et al., 2013; Hu et al., 2014) has been shown to potentially modulate synaptic transmission in a protein synthesis independent manner (Jiao and Li, 2011). Another substrate is involved in regulation of the activity of protein phosphatases (Comerford et al., 2006), which could in turn modulate the phosphorylation levels of other key enzymes such as p38 MAPK (Kitatani et al., 2006). Many indicated interactions need still to be confirmed to take place in the hippocampus, but the few presented examples and our own data point toward protein synthesis independent modulation of cellular processes by eEF2K. However, the specific network of signaling pathway with its final targets requires further studies.

To discover a new branch of the eEF2K signaling pathway that might have been altered by the compound, we tested a variety of key enzymes that have been shown to be involved in activity-dependent synaptic plasticity and more importantly in the regulation of neurotransmitter release (Capogna et al., 1995; Hilfiker et al., 1999). For example, the activation of CaMKII and PKC have been shown to increase vesicle release by the phosphorylation of synapsin-I (Ohyama et al., 2002; Ninan and Arancio, 2004) or Munc-18 (Fujita et al., 1996; Dulubova et al., 1999) respectively. In addition, PKA can also contribute to the vesicle release by phosphorylating RIM1α (Lonart et al., 2003), SNAP-25 (Hepp et al., 2002), Snapin (Thakur et al., 2004) and Syntaphilin (Boczan et al., 2004). However, in our experiments, no effects of specific inhibitors of these kinases on the degree of compound-induced fEPSP potentiation were observed.

A brief rise in the intracellular calcium concentration is known to be a key step for the evoked release of glutamate from presynapses. The origin of the increase in intracellular calcium is manifold and can rely on intracellular calcium release from ER. This mechanism plays an important role in regulating neural function, including the excitability of hippocampal pyramidal cells (Borde et al., 2000; Shah and Haylett, 2000; Shirasaki et al., 2001), and might determine the frequency and amplitude of mEPSCs (Sharma and Vijayaraghavan, 2003) and the degree of presynaptic plasticity (Emptage et al., 2001; Liang et al., 2002; Martin and Buno, 2003). The release of calcium from the ER can be induced by the activation of calcium transporter SERCA (Zucchi and Ronca-Testoni, 1997) or the activation of InsP3 receptors by elevated levels of InsP3. However, depletion of the calcium contents of the ER by thapsigargin or blockage of InsP3 receptors did not prevent the compound-induced potentiation. Only the inhibition of RyRs by dantrolene showed a tendency to attenuate the potentiation of synapses. Interestingly, Reese and Kavalali (2015) reported a critical role of RyRs in amplifying NMDA receptor-driven Ca^2+^ signals at rest for the maintenance of synaptic potentiation.

As discussed above, signaling pathways relevant to classical plasticity are not involved in the compound-mediated effects on synaptic transmission. In addition, we showed that the activity of the PI3K-Akt and MEK/ERK pathways was not required for this synaptic potentiation. Only an inhibitor against the p38 MAPK was shown to prevent the potentiation of synaptic transmission mediated by eEF2K inhibition efficiently. The p38 MAPK is activated by multiple mechanisms, including extracellular stresses, cytokines, growth factors, and osmotic shocks (Cuenda and Rousseau, 2007). The regulation of p38 MAPK also provokes much interest due to its role in synaptic plasticity and neurodegenerative disorders (Correa and Eales, 2012). Indeed, we were able to prove that the inhibition of p38α and p38β by SB203580 (Capron et al., 2006; Kidambi et al., 2010; Jin et al., 2013; Hasegawa et al., 2014) prevents the synaptic potentiation caused by the inhibition of eEF2K. In addition, we showed that the inhibition of eEF2K did not only attenuate the phosphorylation of eEF2 but also significantly increased the phosphorylation level of p38 MAPK within a short time, which was consistent with the results of another study on eEF2K (Usui et al., 2015). However, how the inhibition of eEF2K can cause an enhancement of p38 phosphorylation remains unknown. To date, we have not found any study that addresses a potential interaction of the compound A-494854 with upstream components of the p38 MAPK pathway. A reversed interaction of p38 MAPK on eEF2K has been shown by the p38-dependent phosphorylation of MK2/3 and subsequent phosphorylation of Ser-377 of the eEF2K that slightly inhibited its activity (Knebel et al., 2002).

In the search for potential targets that mediated the synaptic potentiation, we also focused on potassium channels. For example, p38 MAPK regulates HCN gate channels (h-channels; I_h_) in hippocampal pyramidal neurons (Poolos et al., 2006; Jung et al., 2010). However, in our experiments, the inhibition of the h-channel did not prevent the potentiation of synaptic transmission mediated by eEF2K inhibition, either the cesium- and TEA-sensitive potassium channels. Only prevention of the activation of barium-sensitive potassium channels attenuated the effect of eEF2K inhibition on synaptic transmission. A channel that belongs to the two-pore potassium channel family with specific Ba^2+^ selectivity is the TREK-1 channel (Wulff and Zhorov, 2008; Ma et al., 2011). Indeed, blockage of the TREK-1 channel by fluoxetine prevented the eEF2K-inhibition mediated potentiation. TREK-1 channels are expressed both pre- and post-synaptically (Honore, 2007; Westphalen et al., 2007; Sandoz et al., 2009; Veale et al., 2014) and contribute to the maintenance of the neuronal resting potential and membrane excitability by opposing depolarization. In response to its phosphorylation, the outflow of potassium ions is reduced and leads to a slight depolarization of the membrane potential. This feature may account for the effect on the synaptic response by eEF2K inhibition. Since this receptor can be phosphorylated through phosphorylation by PKC and PKA (Murbartian et al., 2005), it may also represent a direct target of p38 MAPK which need further validation. The interpretation of the effects of the compound fluoxetine on eEF2K mediated synaptic responses is complicated by its interference with pathways such as PKC, PKA, or ERK (Edgar et al., 1999; Fumagalli et al., 2005; Stepulak et al., 2008). Nevertheless, we think that we can rule out some of its side effects due to our experiments that excluded the involvement of PKC, PKA, and ERK in the eEF2K-inhibition effect. Further research is required using genetically modified animals or more specific antagonist against TREK-1. With regard to the potential involvement of TREK-1 in neurological diseases, it has been already shown that TREK-1 can be beneficial against mood disorders. Genetic deletion of the background potassium channel TREK-1 creates immunity to depression-inducing paradigms (Heurteaux et al., 2006; Mazella et al., 2010; Borsotto et al., 2015; Vivier et al., 2016). Therefore, these results suggest a possibility to regulate TREK-1 by specific modulation of eEF2K activity.

### Synchronization of Neuronal Network Activity by Overall Regulation of Synaptic Transmission

We showed that A-484954 induced a synchronization of the neural network in primary hippocampal cell cultures. A previous study showed that the regulation of dendrotoxin-sensitive potassium channels contributes to the pattern of neuronal activity (Cudmore et al., 2010). Similarly, our data show the potential implication of barium-sensitive potassium channel TREK-1 in the regulation of neural network activity.

The synchrony of neuronal network activity is an important parameter for physiological function of cognitive abilities (Llinas and Ribary, 2001). Many studies have reported that alteration in the mechanisms of synchrony of neuronal networks could alter the cognitive abilities of schizophrenic and autistic and Alzheimer’s disease patients and can induce motor dysfunctions (Uhlhaas and Singer, 2006). In addition, high levels of p-eEF2 were also reported in a mouse model of Alzheimer’s disease (Li et al., 2005) and in brain material of patients affected by Alzheimer’s disease, suggesting a particular role of the eEF2K/eEF2 pathway in mental disorders. Moreover, the pharmacological and genetic inhibition of eEF2K was recently reported to be able to reverse the epileptic phenotype in a mouse model of human epilepsy (Heise et al., 2016). These data show a novel role of eEF2K in the regulation of brain network activity, providing a novel target for brain disorder treatment.

## Conclusion

**FIGURE 12:**
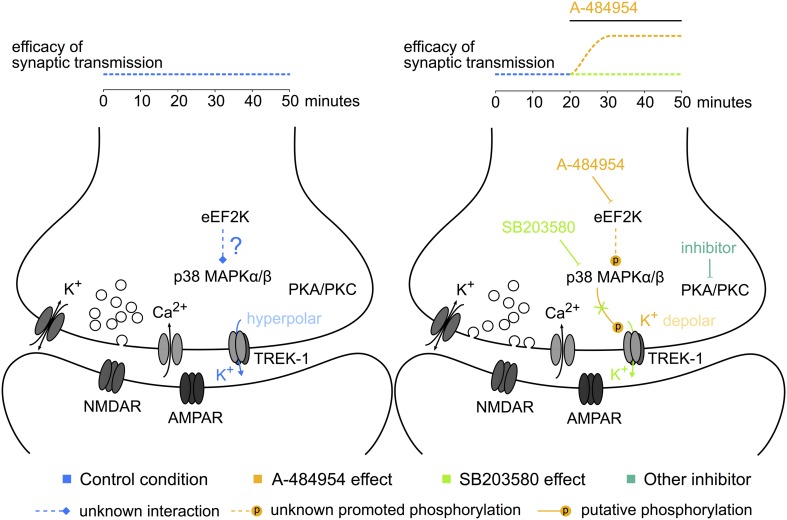
**Potential elements of the eEF2K-inhibition mediated potentiation.** The sketch to the left indicates some of the components studied and their interaction or activity during baseline stimulation. To the right, the modulation of the phosphorylation level and the resulting synaptic potentiation after eEF2K-inhibition are presented. The color of the text corresponds to the potentiation indicated above the sketch. Dotted lines: unknown pathways of interaction; lines ending with a stroke: inhibition; lines ending with a P: phosphorylation. A hypothesis based on our data implicates that the inhibition of eEF2K mediates a potentiation of synaptic transmission by altering the release probability of vesicles with the participation of p38 MAPK and potassium channel phosphorylation-mediated reduction of the resting membrane potential at the synaptic button.

We have presented experimental data concerning a novel role of eEF2K in hippocampal synaptic transmission (**Figure 12**). Further research is required to study whether such compounds could be beneficial for the development of mood disorder treatments with a fast-acting antidepressant response without dissociative psychedelic effects.

## Materials and Methods

### Animals

Sprague Dawley rats (aged 6–10 weeks) were provided from the animal center of the Chinese Academy of Sciences (CAS, Shanghai, China). The animals were maintained in accordance with the established standards of animal care and procedures of the Institutes of Brain Science and State Key Laboratory of Medical Neurobiology of Fudan University, Shanghai, China. Efforts were made to minimize the number of animals studied.

### Electrophysiology

#### Hippocampal Slice Preparation

The preparation of acute hippocampal slices closely followed previously published methodologies (Behnisch et al., 2011; Zhu et al., 2012; Huang et al., 2015). Briefly, after anaesthetization with isoflurane, brains were immersed in ice-cold ACSF (95% O_2_/5% CO_2_ and composition in mM: 124 NaCl, 2.5 KCl, 2.5 CaCl_2_, 2 MgCl_2_⋅6 H_2_O, 1.25 KH_2_PO_4_, 10 glucose, 26 NaHCO_3_, pH 7.3–7.4). Immediately after preparation of the slices, the CA3 area was disconnected from the CA1 region in all experiments to prevent the propagation of spontaneous recurrent activity from CA3 pyramidal neurons. These transverse hippocampal slices (400 μm) were incubated in a submersion-incubation chamber at 30°C for at least 1 h. Electrophysiological experiments were performed in a submerged type recording chamber system (Warner Instruments, RC-26GLP) mounted on a Nikon Eclipse FN1 microscope with constant perfusion (4 mL/min) of ACSF at 30°C.

#### Field Potential Recording

Field excitatory postsynaptic potentials (fEPSPs) were recorded in the stratum radiatum of the hippocampal CA1 area via borosilicate micropipettes filled with ACSF (Huang et al., 2015). Unipolar stimulation electrodes were applied to stimulate Schaffer collateral fibers every minute. Recorded field potentials were amplified by MultiClamp 700B (Axon, USA) and then digitized at a sample frequency of 20 kHz and filtered (2.8 kHz low-pass, 0.1 Hz high-pass). Stimulation strengths were adjusted to 40–50% of the maximum fEPSP slope values. fEPSP slopes or fEPSP amplitudes were determined and expressed as a percentage of baseline values (before drug application).

#### Whole-Cell Voltage-Clamp Recording

Miniature excitatory postsynaptic currents (mEPSCs) of CA1 neurons were recorded using 5–6 MOhm pipettes filled with a solution containing (in mM): cesium methanesulfonate 110, CsCl 10, MgCl_2_⋅6H_2_O 2, EGTA 0.5, ATP-Na_2_ 4, GTP-Na 0.4, phosphocreatine-Na_2_ 10, creatine phosphokinase 50, pH 7.25. Tetrodotoxin (1 μM) and bicuculline (10 μM) were added to ACSF to prevent action potential-driven transmitter release and blockage of GABA_A_ receptors. mEPSCs were amplified by a MultiClamp 700B amplifier, digitalized with Digidata 1440A, and acquired by Clampex 10.2 software (Molecular Devices, Sunnyvale, CA, USA). Membrane potentials were held at -72 mV and currents were passed through a 2.8 kHz Bessel filter. The amplitude and frequency of the mEPSCs were analyzed using MiniAnalysis 6 (Synaptosoft Inc., Fort Lee, NJ, USA). mEPSCs were collected at intervals of 3 min before and 20 min after drug or vehicle application.

#### Drug Treatment

A-484954 (Sigma–Aldrich, SML0861), TTX (Sigma–Aldrich, T8024), Bicuculline (Tocris, 2503), Anisomycin (Sigma–Aldrich, A9789), Cycloheximide (Sigma–Aldrich, C1988), KN-93 (Sigma–Aldrich, K1385), Gö 6983 (Sigma–Aldrich, G1918), KT5720 (Tocris, 1288), Thapsigargin (Sigma–Aldrich, T9033), 2-APB (Sigma–Aldrich, D9754), Dantrolene (Sigma–Aldrich, D9175), U0126 (Sigma–Aldrich, U120), LY294002 (Sigma–Aldrich, L9908), SB203580 (Sigma–Aldrich, S8307), CsCl (Sigma–Aldrich, C3032), TEA (Sigma–Aldrich, T2265), BaCl_2_ (Sigma–Aldrich, 342920), and Fluoxetine (Sigma–Aldrich, F132) were added to ACSF as indicated.

Hippocampal slices were pretreated with these drugs for 1 h, and fEPSPs were recorded in stratum radiatum of CA1 area. A-484954 was applied after at least 20-min stable baseline.

#### Protein Biochemistry

The antibodies and supplier of primary antibodies: rabbit anti-p-eEF2 (Thr56) (1:1000; Cell Signaling Technology: 2331); rabbit anti-p-p38 MAPK (Thr180/Tyr182) (1:1000; Cell Signaling Technology: 4511); rabbit anti-GAPDH (1:1000; Cell Signaling Technology: 2118); rabbit anti-β-actin (1:1000; Cell Signaling Technology: 4970).

Secondary antibodies: horseradish peroxidase-conjugated goat anti-rabbit (1:10000; Jackson ImmunoResearch West Grove: 111-035-003); horseradish peroxidase-conjugated goat anti-mouse (1:10000; Jackson ImmunoResearch West Grove: 115-035-003).

Whole cell proteins were extracted from a 400-μm CA1 region of hippocampal slices using RIPA lysis buffer [Beyotime: Tris (pH 7.4), 150 mM NaCl, 1% Triton X-100, 1% sodium deoxycholate, 0.1% SDS and sodium orthovanadate, sodium fluoride, EDTA, leupeptin] with protease inhibitor (Roche: 04693124001) and phosphatase inhibitor (Roche: 04906837001). After ultrasonication (15 min), the samples were centrifuged (12000 rpm, 5 min) and the supernatant was removed from the pellet, mixed with 4 × SDS loading buffer, boiled for 10 min and then stored at -80°C. Protein concentrations were determined by the bicinchoninic acid method (BCA Protein Assay Kit, Beyotime: P0012S, China). Equal amounts of proteins were separated on an SDS gradient gel and then transferred onto polyvinylidene difluoride membranes (Millipore: IPVH00010). After blocking in 5% fat-free milk for 1 h, the membranes were incubated with primary antibodies overnight at 4°C. On the next day, the membranes were washed and incubated with secondary antibodies. The blots were then incubated with the chemofluorescent reagent ECL (Thermo Scientific, Rockford, IL, USA).

#### Cell Culture

Primary hippocampal neuronal cultures were prepared from P1 old Sprague Dawley rats following standard protocols. The cultures were kept for 2 weeks at 37°C in the 95% O_2_/5% CO_2_ incubator. After 2 weeks, the cultures were transduced with a GCamp6f expressed by the addition of 1 μl non-diluted AAV9 titer.

#### Calcium Imaging

Cover slides with the primary hippocampal cell cultures at DIV 12 were transferred to a submerged type recording chamber system (Warner Instruments, RC-26GLP) 1 week after transduction of the neurons with GCamp6f. The neurons were imaged through a 16x water dipping objective (16x, NA: 0.8; Nikon). Fluorescence proteins were excited by blue light (X-Cite 120LED; Lumen Dynamics Group, Canada). A sequence of frames was acquired for 69.5 s at 46 fps using a PCO.edge 5.5 (PCO, Germany) CMOS camera mounted on a Nikon Eclipse FN1 microscope.

An averaged image over all frames was then subtracted from the individual frames of a sequence (ImageJ). This procedure reduced the appearance of neurons that did not alter their fluorescence over time. Then every remaining neuron was labeled with a region of interest and the maximum gray values for every frame and neuron were calculated. In SPSS, each pair of the neurons were cross-correlated by intensity versus time. The heat map of resulting Pearson’s correlation coefficients for the individual pairs were generated using R programming language. To generate a signal intensity heat map and analog traces, the values were further normalized in GraphPad by the average gray values of the initial 2 s of the sequence (F_0_). The normalized values were plotted as (F-F_0_)/F_0_.

### Statistical Analysis

All data are expressed as mean ± standard error of mean (SEM). The values of different experimental conditions were compared using two-sided paired or non-paired *t*-test. A *p* < 0.05 was considered to indicate a statistically significant difference between two groups. Drug experiments were interspersed with drug-free controls.

## Author Contributions

WW: conducted, analyzed and designed experiments and wrote the manuscript. YC: utilized hippocampal primary cell cultures and calcium imaging. MW: conducted the protein biochemistry. YZ: supported the protein biochemistry, and animal experiments. TB: designed experiments and graphs and wrote the manuscript.

## Conflict of Interest Statement

The authors declare that the research was conducted in the absence of any commercial or financial relationships that could be construed as a potential conflict of interest.
